# Identification of Material Parameters for the Simulation of Acoustic Absorption of Fouled Sintered Fiber Felts

**DOI:** 10.3390/ma9080709

**Published:** 2016-08-22

**Authors:** Nicolas Lippitz, Christopher Blech, Sabine Langer, Joachim Rösler

**Affiliations:** 1 Institute for Materials, TU Braunschweig, Braunschweig 38106, Germany; j.roesler@tu-braunschweig.de; 2 Institute for Engineering Design, TU Braunschweig, Braunschweig 38106, Germany; c.blech@tu-braunschweig.de (C.B.); s.langer@tu-braunschweig.de (S.L.)

**Keywords:** X-ray, acoustic modeling, absorption, fouling, sintered fiber felt, biot

## Abstract

As a reaction to the increasing noise pollution, caused by the expansion of airports close to residential areas, porous trailing edges are investigated to reduce the aeroacoustic noise produced by flow around the airframe. Besides mechanical and acoustical investigations of porous materials, the fouling behavior of promising materials is an important aspect to estimate the performance in long-term use. For this study, two sintered fiber felts were selected for a long-term fouling experiment where the development of the flow resistivity and accumulation of dirt was observed. Based on 3D structural characterizations obtained from X-ray tomography of the initial materials, acoustic models (Biot and Johnson–Champoux–Allard) in the frame of the transfer matrix method were applied to the sintered fiber felts. Flow resistivity measurements and the measurements of the absorption coefficient in an impedance tube are the basis for a fouling model for sintered fiber felts. The contribution will conclude with recommendations concerning the modeling of pollution processes of porous materials.

## 1. Introduction

The constant exposure to noise affects physical health and can cause insomnia. Especially in areas close to airports, this has become a serious problem. Due to a rising number of flights, it is about to become even more serious. Facing this problem, the noise reduction of commercial aircrafts has become important in engineering. One approach to reducing the noise of commercial aircrafts is to use a porous trailing edge. Experimental and numerical results show that this reduces the aeroacoustic noise, generated by flow around the wings of aircrafts. This noise is a major part of the noise generated by flow around the airframe and landing gear and dominating during landing [1,2,3,4]. The functionality of porous materials for noise reduction depends on the porous structure, characterized by porosity, pore size and pore shape. Mounted on trailing edges of airplane wings, the porous materials are exposed to dirt, which is expected to accumulate in the pores during service. This would change the size and shape of the pores as well as the porosity, and, as a consequence, affect the aeroacoustic performance. As the porous material is carefully chosen to reduce the noise of specific phenomena, the affect of dirt must be known. On this basis, the reduced effectiveness can be estimated.

Promising materials for porous trailing edges that achieved a significant reduction of noise in acoustic wind tunnel experiments are sintered fiber felts [5]. To study the influence of dirt accumulation on the acoustic properties of sintered fiber felts, a selection of two different fiber felts was exposed to a long-termed pollution process, and the porous structures were regularly examined over a total period of 12 months. In this paper, the two extremes (zero and twelve months) are compared. Material parameters obtained from X-ray tomography scans are the basis for the application of acoustic models (Biot and Johnson–Champoux–Allard) in the frame of the transfer matrix method. The models are applied to one of the sintered fiber felts. A gradient based optimization algorithm is used to identify missing parameters that are mainly the viscous and the thermal length of the material.

## 2. Fouling Experiment

For the fouling experiment, samples of two different sintered fiber felts, differing in thickness and fiber diameter, were mounted on the roof rack of a car and fouled for a period of up to 12 months. The samples were separated into a set of samples for flow resistivity measurements, consisting of one sample with a size of 120 mm × 120 mm and a set of three samples for structural inspections with a size of 20 mm × 20 mm for every sintered fiber felt. The flow resistivity was measured after one, six and 12 months and compared to the values of the clean samples. At the same time, the smaller samples were used to check the material for fouling and structural changes. Other than for the flow resistivity measurements, a separate sample was used for the three structural analyses during the experiment. After the last flow resistivity measurements, small circular samples were punched out of the bigger samples used for flow resistivity measurements to measure the absorption coefficient using an impedance tube.

## 3. Material Characterization

### 3.1. Structural Characterization

The two different sintered fiber felts (SFF) that were chosen were characterized before the fouling experiments using light microscopy, scanning electron microscopy (SEM) and X-ray tomography. Due to the low density of the organic materials accumulating during the fouling process, X-ray tomography cannot be used to visualize fouling of porous metals. Therefore, only SEM was used to analyze the fouling and changes of porous structure during the 12 months experiment. To ensure the conductivity of the fouled samples, they were sputtered with gold.

#### 3.1.1. Initial Material

**Figure 1:**
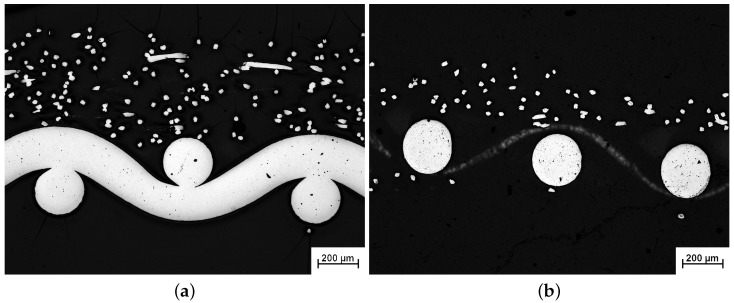
Light microscope images of Sintered Fiber Felts (SFF) before fouling. (**a**) SFF 100; (**b**) SFF 150.

**Figure 2:**
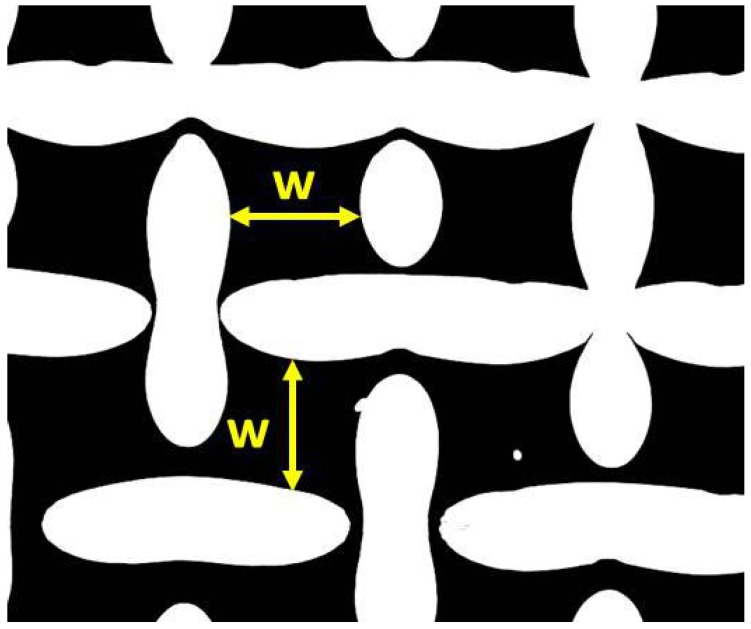
Plane in support grid of SFF 100 extracted from 3D reconstruction.

The sintered fiber felts were received from “GKN Sinter Metals” (GKN Sinter Metal Filters GmbH, Radevormwald, Germany) and consist of a functional layer and a support grid to ensure mechanical stability. The two different fiber felts differ in thickness of the functional layer, whereas the fiber diameter and porosity of the functional layer are similar. The light microscope images in Figure 1 show cross sections of the sintered fiber felts SFF 100 and SFF 150 used for this study. Structural parameters for the functional layers of the clean fiber felts were determined in [6,7] using three-dimensional CT (Computed Tomography) scans and can be found in Table 1. The support grid of both fiber felts is a plain weave with square mesh. The mesh size is the same in the directions of warp and shut wires and indicated as *w*. It was measured from 2D planes extracted from 3D reconstructions (compare Figure 2). As warp and shut wire are very similar in diameter, an averaged wire diameter $d_{w}$ is used for this work. The mesh size *w* and wire diameter $d_{w}$ were used to calculate the porosity $\phi_{w}$ as well as a hydraulic radius $R_{h}$ of the support grid [8,9,10]:

(1) $$\phi_{w}=1-\frac{\pi }{4}\frac{d_{w}\sqrt{d_{w}^{2}+{\left(w+d_{w}\right)}^{2}}}{{\left(w+d_{w}\right)}^{2}}$$

(2) $$R_{h}=1\sqrt{\phi_{w}{\left(w+d_{w}\right)}^{2}}$$

The structural parameters of the support grids, measured and calculated, can be found in Table 1. Compared to the functional layer, the pores of the support grid are significantly bigger. Therefore, the acoustic properties are expected to be mainly determined by the functional layer.

#### 3.1.2. Fouled Material

**Figure 3:**
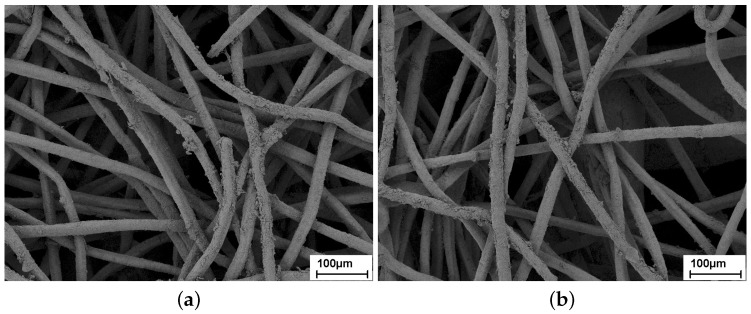
SEM images of sintered fiber felts after 12 months of fouling. (**a**) SFF 100; (**b**) SFF 150.

Due to the low density of the dirt, accumulated in the sintered fiber felts, only SEM could be used to examine the fouled samples. Furthermore, the preparation of cross-sectional samples could have altered the fouling pattern through cutting and embedding of the samples. Therefore, only top view images of the functional layer were used. Images of SFF 100 and SFF 150 after 12 months fouling are shown in Figure 3. Both fiber felts show a thin layer of dirt on the fibers. Particles with a size of several micrometers, which could block pores and have a significant impact on the flow resistivity, were not found. Of SFF 100, only the fibers close to the surface are visible in the top view, whereas for SFF 150, which has a significantly thinner functional layer, wires of the support grid are visible through the functional layer. For SFF 100, dirt that blocks pores and influences the flow resistivity could be accumulated deeper in the functional layer, where it cannot be seen from the surface. For SFF 150, a significant impact of pollution in the functional layer on the flow resistivity is not expected.

### 3.2. Acoustic Characterization

The flow resistance *R* and the absorption coefficient α(f) of the specimens are measured before and after the long-term pollution experiment. The flow resistance *R* is measured using the alternating air flow method as standardised in [11]. Table 2 shows the calculated specific flow resistance $R_{S}$ for clean and fouled sintered fiber felts [11]:

(3) $$R_{S}=RA$$

where *A* is the area of the specimen. The flow resistivity $r=R_{S}/t_{f}$ (length related flow resistance, also shown in Table 2) is related to the thickness of the functional layer $t_{f}$. The reason is a significantly lower flow resistivity of the support grid, which is assumed to be negligible for the measurement. As a material parameter, it is suitable to compare sintered fiber felts with different thicknesses and will be used for the following observations.

Due to fouling, the flow resitivity of SFF 100 is increased by 8.4%. In contrast, *r* of SFF 150 is only slightly increased by 2.3%. This corresponds to the observations made in SEM images of the fouled samples.

A measurement of the support grid only was not possible due to the lower limit of the measurement equipment. Hence, the flow resistivity of the support grid is calculated according to [12], assuming cylindrical pores with circular cross-section and using the hydraulic radius from Table 1:

(4) $$r=\frac{8\eta }{R_{h}^{2}\Phi_{w}}=2613\;\mathrm{Ns}/m^{4}$$

For $R_{h}$ and $\Phi_{w}$, values from Table 1 are used.

**Figure 4:**
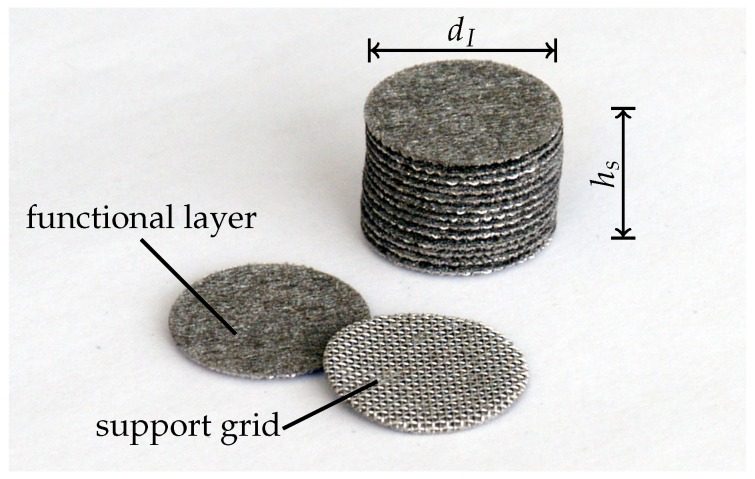
Punched specimens of SFF 100.

The frequency-dependent absorption coefficient α(f) is received applying the standardised method described in [13]. An impedance tube of diameter $d_{I}=0.019\;$m is available for the measurement, which results in a lower frequency limit of $f_{l}=1140\;\mathrm{Hz}$ and an upper frequency limit of $f_{u}=10,400\;\mathrm{Hz}$ [13]. This frequency range is of practical relevance and showed a significant reduction of noise in acoustic wind tunnel experiments [2,3,5]. Previous work [14] shows that the applied impedance tube has a sufficient accuracy up to 8000Hz in practice. Hence, the upper limit of investigation is additionally decreased to $f_{u}=8000\;$Hz. The functional layer of the SFF is oriented facing the planar acoustic waves. The specimens are punched out of the square cuts of the polluted samples and stapled for the measurement (see Figure 4).

Due to the small thickness of 0.0007to0.0009m, a measurement of a single layer is not reasonable. The staple sizes are documented in Table 3. Due to a slight skewness of the specimens and inhomogeneities concerning the thicknesses, the number of specimens does not ideally fit to the staple sizes. Within the experiment, the number of specimens is counted and the total staple size is measured.

**Figure 5:**
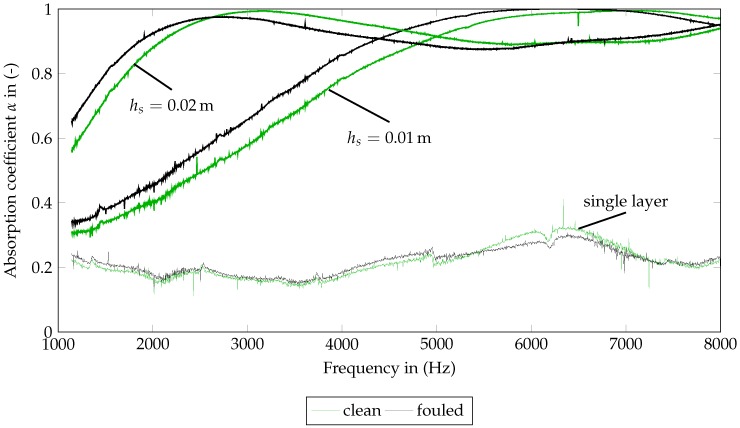
Measured frequency-dependent absorption coefficients of clean (0 M) and fouled (12 M) SFF 100 for one layer and staple sizes of 0.01m and 0.02m.

**Figure 6:**
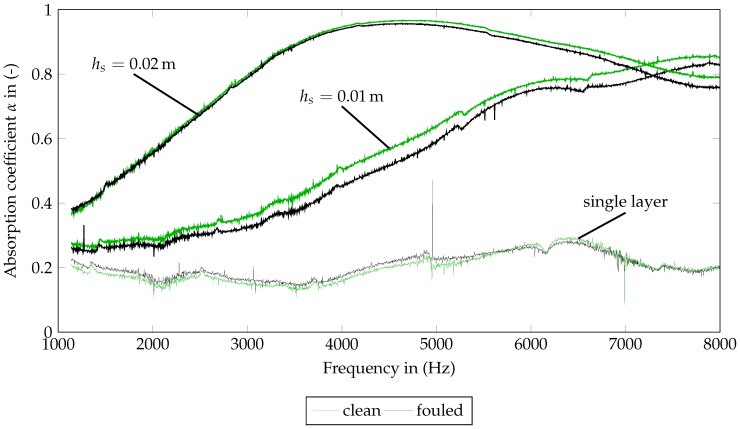
Measured frequency-dependent absorption coefficients of clean (0 M) and fouled (12 M) SFF 150 for one layer and staple sizes of 0.01m and 0.02m.

The associated results for the frequency-dependent absorption of SFF 100 and SFF 150 coefficient can be seen in Figure 5 and Figure 6. For the measurement of a single layer, both figures point out noise dominating the measurement. A material characterization on that basis is not reasonable.

The SFF 100 (Figure 5) shows different absorption curves for staple sizes $h_{s}$ of 0.01 m and 0.02m. The first maxima with absorption coefficients close to one are located at 7000Hz and 3200Hz, respectively, for $h_{s}=0.01$ m and 0.02m. The absorption curve of the largest staple size ($h_{s}=0.02\;$m) shows the characteristic wave after the fist maximum. In accordance with [12], this zone should be covered to ensure an appropriate parameter identification of the material’s characteristic lengths. Hence, this staple size is chosen for the numerical investigation in Section 4.

The absorption curves of the fouled SFF 100 are, for the most part, slightly shifted to lower frequencies and slightly decreased for $h_{s}=0.02\;$m. As indicated in Section 3.1, the effect of the fouling process is small.

The SFF 150 (Figure 6) shows a similar behavior to the SFF 100. For the clean SFF 150, the first maximum can only be identified at 4800Hz for $h_{s}=0.02\;$m. Consistent with the small change in the flow resistivity, the absorption curve of the fouled SFF 150 is hardly distinguishable from the curves of the clean SFF 150. Concerning the flow resistivity and the absorption, this material seems to be quite resistant to the pollution experiment. Therefore, in the modeling part (Section 4), the focus is laid on the SFF 100.

## 4. Numerical Investigation

As mentioned in Section 3.2, this part focuses on the numerical investigation of clean and fouled SFF 100. The aim is a modeling of the frequency-dependent absorption coefficient α(f) for normal incidence to inversely receive the material’s unknown parameters. The application of these parameters may help to understand fouling processes in more complex porous structures. The unknown parameters are fitted using an in-house implementation of the Transfer Matrix Method (TMM) in combination with a gradient-based optimization algorithm in MATLAB (R2013a, Natick, MA, USA) . The TTM is proposed in [15] as a general method to calculate the response of a infinite extended layered structure excited by a plane wave. For each layer, a transfer matrix is calculated to describe the wave propagation from one face to the other. The following material models are introduced to achieve the layer matrices and form the system matrix. The velocity and pressure of the layered structures are the results. On that basis, the absorption coefficient *α* of the stapled system can be derived. The in-house implementation is verified including the coupling between different material models by use of results from literature [12].

### 4.1. Modeling of Stapled Sintered Fiber Felts

The functional layer and the support grid of the SFF 100 material are modeled using different approaches. The response of the functional layer is modeled using Biot’s model [16]. This approach considers waves within the structural and fluid phases of the functional layer. As effects in the fluid phase of the support grid on the overall absorption coefficient are expected, the grid is modeled using the Johnson–Champoux–Allard model [17,18]. Here, structural waves are neglected and the grid itself is treated as rigid perforated screen. The parameters needed for the two models are mainly taken from the structural and acoustic characterizations (Section 3.1 and Section 3.2) and given in Table 4.

Concerning the functional layer, the unknown parameters are the tortuosity $\alpha_{\infty }$, the viscous characteristic length Λ and the thermal characteristic length $\Lambda^{\prime }$.

- **Tortuosity** $\alpha_{\infty }$ is defined by Johnson in 1987 [17]. The value describes the relationship between the squared macroscopic and microscopic velocity to calculate an effective density of the fluid. For common fiber felts, tortuosity is expected near unity [19].
- **Viscous characteristic length Λ** is introduced by Johnson et al. as an extension of the hydraulic radius $R_{H}$ (equal with factor 2 for cylindrical pores) in 1986 [20]. The value is defined as two times the weighted pore’s volume integral divided by the weighted pore wall’s surface integral. The weighting is realised by the fluid’s velocity, respectively, within the volume and on the surface. For common porous materials, the following assumption is taken from literature [21]:
  (5) $$\Lambda =\frac{1}{c}{\left(\frac{8\alpha_{\infty }\eta }{R_{h}\Phi }\right)}^{0.5},\;0.3\leq c\leq 3.3$$
  where *c* is an acoustic value describing the pores and *η* is the dynamic viscosity of air ($\eta (25{\;}^{\circ }C)=1.86e-5\;\mathrm{Pas}$).
- **Thermal characteristic length** $\Lambda^{\prime }$ is defined by Champoux and Allard in 1991 [18] and is used to describe thermal dissipation effects at higher frequencies. $\Lambda^{\prime }$ equals two times the pore’s volume divided by the pore wall’s surface and influences the bulk modulus of the material layer. For common porous materials, the following assumptions are taken from literature [21]:
  (6) $$\begin{array}{ccc} \Lambda^{\prime } & > & \Lambda \end{array}$$
  (7) $$\begin{array}{ccc} \Lambda^{\prime } & = & \frac{1}{c^{\prime }}{\left(\frac{8\alpha_{\infty }\eta }{R_{h}\Phi }\right)}^{0.5},\;0.3\leq c^{\prime }\leq 3.3 \end{array}$$

The assumptions considered for the Biot layer modeling the fiber felt lead to the parameters and parameter ranges shown in Table 4. For sintered fiber felts, the characteristic thermal length $\Lambda^{\prime }$ is expected to be twice as large as the characteristic viscous length Λ [21].

Concerning the support grid, the unknown parameters are the same three as described above. In the case of the coarse grid (see Figure 1), acoustic waves traveling straight through the grid are expected. Hence, the tortuosity is set equal to one. Furthermore, as the grid is assumed to be a macroscopic regularly patterned fibrous material, the following formula are considered for the characteristic lengths [21]:

(8) $$\begin{array}{ccc} \Lambda & = & \frac{1}{2\pi LR_{h}} \end{array}$$

(9) $$\begin{array}{ccc} \Lambda^{\prime } & = & 2\Lambda \end{array}$$

Again, the corrected value for $R_{h}$ is applied to receive the characteristic lengths of the support grid, which are also shown in Table 4.

**Figure 7:**
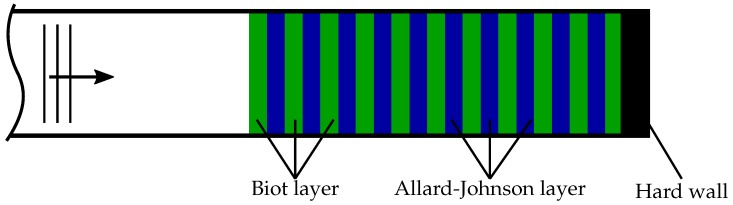
Modeling approach for the acoustic response of stapled specimens.

The two modeling approaches are applied alternating to realize the staples (using TMM) documented in Table 3. This results in a total number of layers $N_{total}=2*N_{s}$. Figure 7 shows a schematic sketch of the alternating layers, finished by a hard wall (total reflection).

### 4.2. Modeling of Fouling

The fouling process of sintered fiber felts leads to dirt located on the fiber’s surface, which may influence the materials’ macroscopic behavior. Due to small particle sizes of the observed dirt, a need for different modeling approaches is not expected. A modification of the model parameters with regard to the fouling process is described as follows.

In Section 3.1, dirt is mainly identified within the functional layer’s microstructure. Hence, the parameters for the Johnson–Champoux–Allard model applied to the layer of the support grid are assumed to be equal before and after the fouling process.

The parameters of Biot’s model applied to thefunctional layer are expected to change due to the fouling process. Mechanical parameters of the dirt are not known and the dirt is expected to have no influence on the structural phase of Biot’s model. The parameters concerning the fluid phase are modified only. In Table 2, the slightly increased flow resistivity is already shown (8.4%) and can easily be applied for the modeling of the fouled SFF 100. Furthermore, in [22], a pollution parameter is adopted and referred to the flow resistivity:

(10) $$\begin{array}{ccc} r_{fouled} & = & \frac{r_{clean}}{{\hat{\Phi }}^{2}} \end{array}$$

(11) $$\begin{array}{ccc} \hat{\Phi } & = & \sqrt{\frac{r_{clean}}{r_{fouled}}} \end{array}$$

(12) $$\begin{array}{ccc} \hat{\Phi } & = & 0.96 \end{array}$$

where $\hat{\Phi }$ describes the relative volume of fluid which is not polluted. In this case, $1-\hat{\Phi }=4$% of the fluid volume is taken by dirt. A new porosity for the fouled material can be calculated [22]:

(13) $$\Phi_{fouled}=\hat{\Phi }\Phi_{clean}=0.88$$

The tortuosity $\alpha_{\infty }$ and the characteristic lengths Λ and $\Lambda^{\prime }$ are assumed to be equal for clean and fouled sintered fiber felts. The reason is that the observed small particles of dirt should not change these parameters significantly.

### 4.3. Inverse Parameter Identification

Applying the received parameters (Table 4) for the clean SFF 100 results in a model with three unknown parameters of the Biot layer (functional layer). The following parameters are found using the gradient based optimization algorithm:

(14) $$\begin{array}{ccc} \alpha_{\infty } & = & 1.3 \end{array}$$

(15) $$\begin{array}{ccc} \Lambda & = & 27\;µ\text{m} \end{array}$$

(16) $$\begin{array}{ccc} \Lambda^{\prime } & = & 27\;µ\text{m} \end{array}$$

As mentioned in Section 4.1, concerning the functional layer, the characteristic thermal length $\Lambda^{\prime }$ is expected to be twice as large as the characteristic viscous length Λ [21]. Equality of these parameters is rather expected for straight cylindrical pores [21]. Here, the equality may be explained by the small thickness of the functional layers, each followed by the support grid with significantly larger pores. This may result in a straight transmission of sound waves within the investigated frequency range, similar to cylindrical pores. A tortuosity of 1.3 is near unity, and, therefore, a realistic value for the SFF material. Due to the coupling of 21 double-layers, rotations of the support grids towards each other may result in a slightly increased tortuosity. An additional investigation of separated support grids would help to verify this statement.

**Figure 8:**
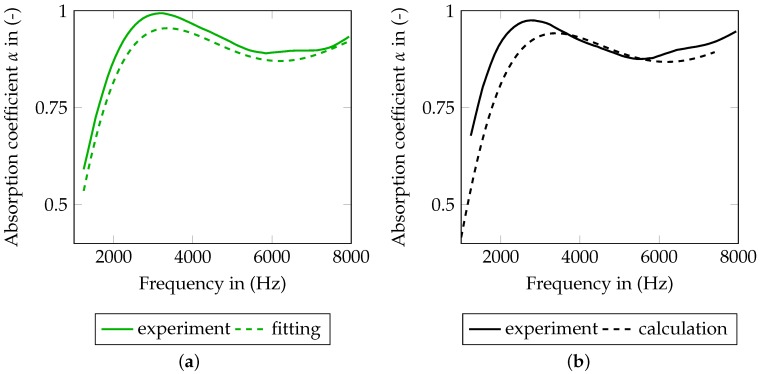
Comparison of numerical and experimental data for a staple size of 0.02m. (**a**) fitting of clean SFF 100; (**b**) calculation (forward solution of the mechanical problem, without any model updating) of fouled SFF 100.

Figure 8a shows the comparison of the measured and fitted curve of α(f). In all plots, measurement data is drawn by the use of solid lines and simulation data with dashed ones. Furthermore, the curves of clean SFF are green and the curves of fouled SFF are black. The curves in Figure 8 are not fitting perfectly. Several reasons are assumed to have a major impact on the results. First, the impedance tube has a relatively high inherent absorption. This may result in raised absorption curves in the measurement. Second, the flow resistance is measured using the whole specimen (before punching). Inhomogeneities may lead to the fact that better absorbing parts are located closer to the end of the staple, which may result in higher absorption. Furthermore, including the support grid in the measurement, a slightly raised flow resistance is measured and finally referred to the functional layer. A separation of the layers within the measurement is not possible.

**Figure 9:**
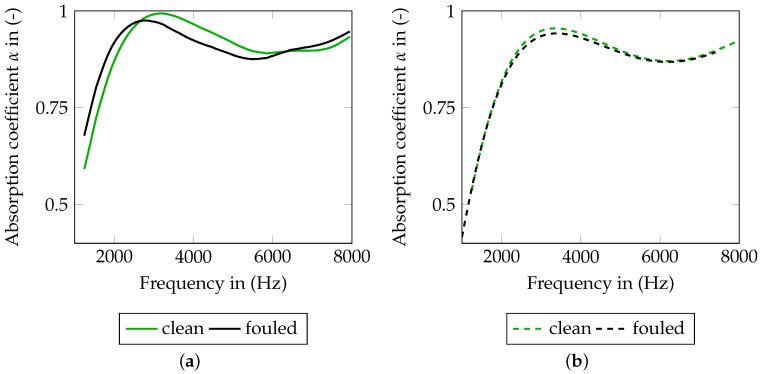
Comparison of clean and fouled SFF 100 for a staple size of 0.02m. (**a**) experimental data; and (**b**) numerical data.

The directly calculated response of the fouled SFF 100 can be seen in Figure 8b. Due to only small changes of *r* and Φ, the absorption coefficient does not change significantly. The shift of the measured curve to lower frequencies cannot be reproduced properly. Figure 9 additionally shows the difference of the measured absorption curves (a) and the calculated absorption curves (b) applying the fouling model. Only a slight tendency of a decreased absorption coefficient is recognizable in the calculation.

To give first starting points for further investigations of the indicated problems concerning multilayered material configurations with several couplings between small layers and the modeling of fouling, an inverse parameter identification of the fouled material is done. $\alpha_{\infty }$, Λ and $\Lambda^{\prime }$ of the Biot layer are fitted using the gradient-based optimization algorithm:

(17) $$\begin{array}{ccc} \alpha_{\infty } & = & 1.5 \end{array}$$

(18) $$\begin{array}{ccc} \Lambda & = & 26\;µ\text{m} \end{array}$$

(19) $$\begin{array}{ccc} \Lambda^{\prime } & = & 26\;µ\text{m} \end{array}$$

**Figure 10:**
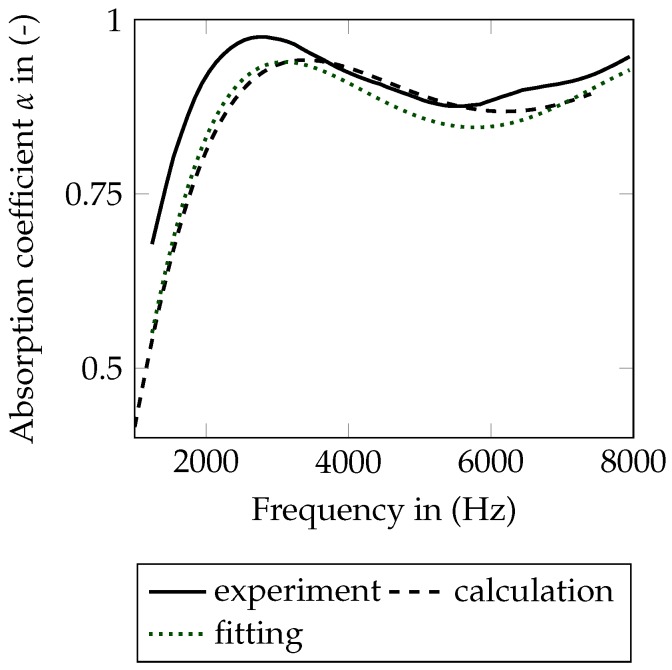
Calculated and fitted curves for fouled SFF 100.

Furthermore, the tortuosity of the support grid was included into the fitting, but the optimizer only changes this value by the amount of 0.2%. Apparently, the introduced degrees of freedom in the functional layer tend to cause an increase of the tortuosity after the pollution process (1.3→1.5). Λ and $\Lambda^{\prime }$ are not changed significantly in comparison to the fitting of the clean SFF 100. Figure 10 shows the better fitting curve with the maximum shifted to lower frequencies. Nevertheless, as already stated for the very first fitting of the clean SFF 100, the absorption coefficient is slightly lowered over the complete frequency range.

## 5. Conclusions

In this paper, two types of sintered fiber felts, underlying a fouling process, are investigated concerning the material parameters. In the long-term fouling experiment, the flow resistivity of the sintered fiber felts increases slightly. This corresponds to the rare pollution observed in SEM images. The frequency-dependent absorption coefficient is slightly shifted to lower frequencies and decreased. If this type of porous material is considered in aircraft applications, only small reductions of the performance are expected. Further study must be carried out to ensure a secure statement on the pollution effects in different use cases.

Within a modeling approach on the basis of Biot’s theory, the two layers of the SFF 100 material are considered. The multilayered model of the clean material predicts the characteristic shape of the absorption coefficient with a small systematic error. The effect of the fouling process can also be predicted qualitatively applying a simple model. Besides an increase of the flow resistivity and a decrease of the porosity, a changing tortuosity should be considered as well. Further investigation should focus on coupling effects in multilayered material configurations and the development of more detailed modeling of fouling.

## Figures and Tables

**Table 1:** Structural parameters of sintered fiber felts in clean condition. Porosity $\phi_{f}$ of the functional layer, thickness of the functional layers $t_{f}$, fiber diameter in the functional layer $d_{f}$, wire diameter of the support grid $d_{w}$, mesh size of the support grid *w*, porosity of the support grid $\phi_{w}$, hydraulic radius of the support grid $R_{h}$.

Material	$\phi_{f}$	$d_{f}$ (μm)	$t_{f}$ (μm)	$d_{w}$ (μm)	*w* (μm)	$\phi_{w}$	$R_{h}$ (μm)
SFF 100	0.92	430	26	254	432	0.69	142
SFF 150	0.94	270	24	252	424	0.69	140

**Table 2:** Specific flow resistance, thickness and flow resistivity of clean and fouled SFF 100 and SFF 150.

Parameter	SFF 100		SFF 150	
	Clean	Fouled	Clean	Fouled
$R_{S}$ (Ns/m${}^{3}$)	51.26	55.56	15.13	15.48
$h_{f}$ (m)	0.00043	0.00043	0.00027	0.00027
*r* (Ns/m${}^{4}$)	119,208	129,209	56,036	57,333

**Table 3:** Staple sizes of punched specimens of SFF 100 and SFF 150.

Material	Staple Size $h_{s}$ (m)	Number of Specimens $N_{s}$ (-)
SFF 100	0.00090.010.02	11121
SFF 150	0.00070.010.02	11225

**Table 4:** Material parameters used for the acoustic models.

Parameter	Symbol	Unit	Functional Layer	Support Grid
layer thickness	$h_{S}$	(m)	0.00043	0.00047
Young’s modulus	*E*	(N/m^2^)	$2\cdot 10^{11}$	-
Poisson ratio	*ν*	(-)	0.3	-
structural density	$\rho_{S}$	(kg/m^3^)	8000	-
loss factor	$\eta_{l}$	(-)	0.01	-
fluid density	$\rho_{F}$	(kg/m^3^)	1.21	1.21
speed of sound	$c_{F}$	(m/s)	343	343
porosity	Φ	(-)	0.915	0.691
flow resistivity	*r*	(Ns/m^4^)	119,208	2610
**tortuosity**	$\alpha_{\infty }$	(-)	1–1.6	1
**viscous length**	Λ	(µm)	11to123	409
**thermal length**	$\Lambda^{\prime }$	(µm)	11to123	204

## Abbreviations

The following abbreviations are used in this manuscript:

CT	Computed Tomography
SEM	Scanning Electron Microscope
SFF	Sintered Fiber Felt
TMM	Transfer Matrix Method
